# Cost Effectiveness of First-Line Treatment with Doxorubicin/Ifosfamide Compared to Trabectedin Monotherapy in the Management of Advanced Soft Tissue Sarcoma in Italy, Spain, and Sweden

**DOI:** 10.1155/2013/725305

**Published:** 2013-11-03

**Authors:** Julian F. Guest, Monica Panca, Erikas Sladkevicius, Nicholas Gough, Mark Linch

**Affiliations:** ^1^Catalyst Health Economics Consultants, 34b High Street, Northwood, Middlesex HA6 1BN, UK; ^2^School of Biomedical Sciences, King's College, London SE1 1UL, UK; ^3^Palliative Care Department, Royal Marsden Hospital, London SW3 6JJ, UK; ^4^Sarcoma Unit, Royal Marsden Hospital, London SW3 6JJ, UK

## Abstract

*Background*. Doxorubicin/ifosfamide is a first-line systemic chemotherapy for the majority of advanced soft tissue sarcoma (ASTS) subtypes. Trabectedin is indicated for the treatment of ASTS after failure of anthracyclines and/or ifosfamide; however it is being increasingly used off-label as a first-line treatment. This study estimated the cost effectiveness of these two treatments in the first-line management of ASTS in Italy, Spain, and Sweden. *Methods*. A Markov model was constructed to estimate the cost effectiveness of doxorubicin/ifosfamide compared to trabectedin monotherapy, defined as the cost per QALY gained, in each country. *Results*. First-line treatment with doxorubicin/ifosfamide resulted in lower two-year healthcare costs and more QALYs than first-line treatment with trabectedin monotherapy in all three countries. Probabilistic sensitivity analysis showed that at a cost per QALY threshold of €35,000, >90% of a cohort would be cost effectively treated with doxorubicin/ifosfamide compared to trabectedin monotherapy in all three countries. *Conclusion*. Within the model's limitations, first-line treatment of patients with ASTS with doxorubicin/ifosfamide instead of trabectedin monotherapy affords a cost-effective use of publicly funded healthcare resources in Italy, Spain, and Sweden and is therefore the preferred treatment in all three countries. These findings support the recommendation that trabectedin should remain a second-line treatment.

## 1. Introduction

Soft tissue sarcomas are a heterogeneous group of rare malignant tumours originating from connective tissue [1, 2] which account for approximately 1% of all adult cancers [3]. Their incidence in the European population is 3 to 4 new cases per 100,000 which has remained stable over time [3]. The risk of developing soft tissue sarcoma increases with age and the disease mostly develops in people over 50 years [4]. Soft tissue sarcomas commonly occur in the extremities (50% of patients), trunk/retroperitoneum (40%), or the head and neck (10%) [5]; they generally develop without pain and can be difficult to diagnose. Prognosis depends on several factors, including patients' age and the size, depth, histologic grade, and stage of the tumour [2, 6]. Curative treatment largely consists of radical surgery and/or radiotherapy. However, these tumours are often aggressive and over 50% of soft tissue sarcoma patients develop metastases [7, 8].

Patients with advanced soft tissue sarcoma (ASTS) present with either locally advanced “inoperable” or metastatic disease [9]. With some exceptions, patients with ASTS are generally considered incurable and have poor long-term survival. Moreover, histological subtypes differ in their sensitivity to cytotoxic drugs [10]. Consequently, patient selection for an appropriate treatment strategy requires expert multidisciplinary team involvement [11, 12].

Palliative chemotherapy is the mainstay of treatment for ASTS where the aim is to establish disease control and improve both quantity and quality of life. Sarcomas have proved resistant to many conventional cytotoxic therapies with only doxorubicin and ifosfamide showing significant response rates when used alone or in combination as first-line treatments [13]. However, high-dose ifosfamide is associated with an increased risk of toxicity [14–16]. Consequently, many clinicians do not initiate chemotherapy with ifosfamide monotherapy. A standard dose combination of doxorubicin and ifosfamide leads to a higher response rate than when either is used as a single agent [17].

Trabectedin is a newly licensed chemotherapeutic agent for the treatment of ASTS, with demonstrable clinical response and an acceptable toxicity profile [18–20]. It is indicated for the treatment of adult patients with ASTS (1) after failure of anthracyclines and ifosfamide or (2) who are unsuited to receive these agents. However, trabectedin is being increasingly used off-label as a first-line treatment. Trabectedin has a relatively high acquisition cost compared to doxorubicin and ifosfamide. In the context of limited healthcare resources, pharmacoeconomic analyses are important in aiding policy makers and clinicians to make the most appropriate decisions about resource allocation and patient management. Against this background, the objective of this study was to estimate the cost effectiveness of doxorubicin/ifosfamide compared with trabectedin monotherapy in the first-line management of ASTS in Italy, Spain, and Sweden from the perspective of the publicly funded health service in each country.

## 2. Methods

### 2.1. Data Sources

A systematic literature search was performed using the search term of ASTS plus one of the following: incidence, prevalence, epidemiology, doxorubicin or Adriamycin and/or ifosfamide, liposomal doxorubicin or Caelyx, ifosfamide and epirubicin, trabectedin or ecteinascidin-743, gemcitabine and/or docetaxel, gemcitabine and dacarbazine, gemcitabine and vinorelbine, gemcitabine and paclitaxel, trofosfamide and/or etoposide, CYVADIC or cyclophosphamide and vincristine and Adriamycin and dacarbazine, utilities, quality of life, cost effectiveness, cost utility, resource utilisation, and economics and cost. The search strategy was not limited by year of publication; English, Italian, Spanish, and Swedish language papers were included. A manual literature search was also undertaken, based on citations in the published papers.

The search included studies published between 1988 and 2010 and included prospective and retrospective studies, randomised and nonrandomised studies, multicentre trials, single centre reports, and clinical reviews. Publications that only reported outcomes for specific subtypes of ASTS were excluded. The review yielded 53 different studies providing data on 2,977 patients. Analysis of the publications provided an estimate of

- the probability of patients achieving complete response (CR), partial response (PR), stable disease (SD), and progressive disease (PD),
- the median duration of each type of response,
- survival rates,
- the incidence of grades 3-4 haematological complications including the incidence of anaemia, febrile neutropenia, neutropenia, and thrombocytopenia.

The literature search was unable to find any health economic studies on ASTS in Italy, Spain, or Sweden. Hence, estimates of healthcare resource use were obtained by interviewing six oncologists in each country who treated patients with sarcoma. The interviews used a structured questionnaire and focused on patient management and resource utilisation.

### 2.2. Economic Model

**Figure 1:**
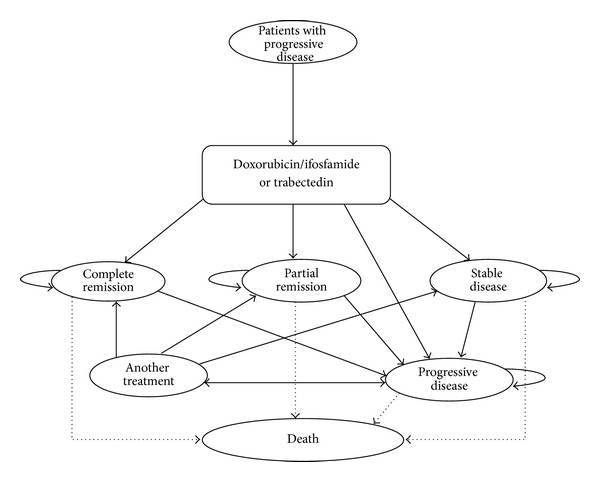
Markov model depicting the management of ASTS in Italy, Spain, and Sweden.

A Markov model was constructed depicting the management of a 65-year-old patient with ASTS (Figure 1). The model spans a period of 2 years and comprises the following health states: progressive disease, (PD), stable disease (SD), partial response (PR), complete response (CR), and death. The model comprises monthly cycles and the arrows depict the possible movement of patients between the different health states.

All patients enter the model with PD and receive treatment with either doxorubicin/ifosfamide or trabectedin. Within the model, following first-line chemotherapy, patients can remain in the PD health state, move into one of the other three health states (i.e., CR, PR, or SD), or die. Patients remain in the CR, PR, and SD health states for the median duration of response, before moving to the PD health state. The model assumed that patients who remain in the PD health state would be switched to a second-line chemotherapy after three cycles of their first-line treatment.

After second-line chemotherapy, patients can again remain in the PD health state, move into one of the other three health states, or they can die. The model only considered first- and second-line chemotherapies. Therefore, following failure of second-line chemotherapy, patients with disease progression were assumed to only receive palliative care alone.

Within the model, patients in any health state can die from age-related factors in accordance with the background death rate. Additionally, patients in the PD health state can die from ASTS-related factors.

#### 2.2.1. Model Inputs: Resource Use

No publications were identified that quantified healthcare resource use for the management of ASTS in Italy, Spain, or Sweden. Therefore, this was estimated using information obtained from interviews with six oncologists in each country who managed ASTS and who collectively saw ~250, 300, and 200 patients with ASTS in Italy, Spain, or Sweden, respectively, at any one time.

*Diagnosis*. New cases of ASTS are generally diagnosed by oncologists, but patients are managed by a multidisciplinary team comprising oncologists, surgeons (general, orthopaedic, or thoracic depending on the site of the tumour), radiation oncologists, pathologists, and any other secondary care specialist depending on the sub-type of ASTS. Diagnosis of ASTS generally takes 2–6 weeks. However, the diagnosis can be delayed by up to 6 months due to unsuccessful biopsies.

According to the interviewees, patients would be seen on an outpatient basis and would have a mean of 3 visits before a diagnosis of ASTS is confirmed. The tests and procedures performed during the diagnostic phase depend on the site of the disease and the histological sub-type of sarcoma. Nevertheless, all patients would have a full clinical examination and undergo the following diagnostic procedures: blood tests (100% of patients), other nonspecified pathological tests (100% of patients), biopsy (50–100% of patients), computerized tomography (CT scan; 75–100% of patients), magnetic resonance imaging (MRI; 40–80% of patients), positron emission tomography (PET scan; 5–35% of patients), chest X-ray (10–20% of patients), and ultrasound scan (5–50% of patients). Also, patients would be assessed for their performance status using the Eastern Cooperative Oncology Group (ECOG) scales and criteria, with regard to disease progression and its influence on patients' daily living abilities [21].

*Treatment*. Patients with ASTS often have widespread metastases and are therefore treated with systemic chemotherapy. Oncologists generally initiate chemotherapy at a mean of 2 weeks (range: 1–4 weeks) following a diagnosis of ASTS. Chemotherapy regimens are tailored according to the type of primary tumour since different sarcoma subtypes respond differently to different drugs. According to the interviewees, up to 75% of patients are expected to receive first-line doxorubicin/ifosfamide. The probabilities of receiving a second-line treatment following a lack of response or disease progression, as estimated by the interviewees and incorporated in the model, are summarised in Table 1.

It has to be noted that treatment patterns identified during the clinician interviews are only indicative, since a significant proportion of patients would be enrolled in clinical trials or only managed with palliative care following treatment failure.

There are no established third-line treatments for ASTS, and any chemotherapy drug that has not been used along the treatment pathway could be used as a third-line treatment and subsequently. Third-line treatments depend on many factors, including previous treatments, the patients' ECOG performance status, their preferences, the histological sub-type of sarcoma, and the level of tolerable toxicity. Consequently, only second-line treatments have been modelled in the present study. Patients who remain alive following failure of a second-line treatment were assumed to only receive palliative care.

The characteristics of all the chemotherapy regimens utilised by the interviewees that have been incorporated in the model are summarised in Table 2.

*Evaluation of Response to Chemotherapy*. Patients generally receive 2–4 cycles of chemotherapy before evaluation of response. This would be ascertained using laboratory tests (100% of patients), CT scan (60–100% of patients), MRI scan (20–40% of patients), PET scan (5–15% of patients), ultrasound (10% of patients), and chest X-ray (8% of patients). Patients may also undergo other tests as needed. Patients not responding to treatment would be switched to a second-line treatment following the first response evaluation and they would be evaluated after another 2-3 cycles. Patients who respond to treatment would continue on it for a mean of 6 cycles or in some cases until disease progression. Nevertheless, an average patient would receive a mean of 6 cycles.

*Pre- and Postchemotherapy Tests*. All patients receiving chemotherapy would undergo haematological and renal function tests before each cycle of chemotherapy. Additionally, patients receiving doxorubicin-containing regimens would usually require functional cardiac assessment with an echocardiography (ECHO)/multiple gated acquisition scan (MUGA). Patients receiving trabectedin would also undergo liver function tests. Some clinicians would also perform a CT scan before each cycle of chemotherapy to monitor response. However, this would only be employed in selected patients and it is not a standard practice. Other tests may be performed before the administration of chemotherapy if toxicity is observed. The tests performed would depend on the type of toxicity present.

Approximately 6–30% of patients experiencing haematological toxicity require dose adjustments, which are very individual and depend on a patient's weight, their tolerance levels, and general performance status. Normally, the chemotherapy dose for the next cycle would be reduced by ~23% of a patient's initial chemotherapy dose (range: 18–28%). This applies to all regimens. Any dose reduction lasts for the rest of the treatment unless a patient's performance status significantly improves. According to the interviewees, dose reduction is most likely to be required at the end of a treatment.

*Clinician Visits*. During the period patients receive chemotherapy, an oncologist would see patients every 3-4 weeks. Patients experiencing haematological toxicity might need to be seen more than once during each cycle. Also, patients receiving a cycle over a few days may be seen on each day of the infusion. No other specialists would see patients during the treatment phase. However, other specialists may become involved if needed (e.g., gynaecological sarcomas would require the involvement of a gynaecologist).

Following completion of the chemotherapy phase, patients with complete or partial response would be seen every 3–6 months by oncologists and radiotherapists only. In some cases patients may require closer monitoring. Those with stable disease would be seen anywhere between every 3 weeks and every 3 months by oncologists and radiotherapists.

*Follow-Up Tests and Procedures*. After chemotherapy, patients who have responded would undergo the following procedures/tests during their follow-up: laboratory tests (100% of patients), CT scan (50–100% of patients), MRI scan (30–45% of patients), PET scan (10–15% of patients), chest X-ray (8% of patients), and ultrasound scan (<1% of patients). A range of other tests would be performed as needed. Follow-up procedures and tests would be performed every 3–6 months.

*Pre- and Postchemotherapy Medications*. Generally, all patients would receive medication before each chemotherapy administration with the aim of preventing haematological or nonhaematological toxicities. In Italy, patients would receive an antiemetic such as granisetron (3 mg; 50% of patients) or ondansetron (8 mg; 50% of patients) and a corticosteroid such as dexamethasone (4–16 mg; 100% of patients). In Spain, patients would receive palonosetron (1 mg; 20% of patients), aprepitant (125 mg; 100% of patients in most regimens except those containing trabectedin), granisetron (2 mg; 20% of patients), metoclopramide (30 mg; 20% of patients) or ondansetron (8–24 mg; 20% of patients), dexamethasone (4–20 mg; 100% of patients), and an antihistamine, such as diphenhydramine (150 mg; 100% of patients). Generally, an antihistamine would be only administered in regimens containing paclitaxel, docetaxel, and trabectedin. Patients receiving gemcitabine- and/or dacarbazine-containing regimens would be given a corticosteroid and an antiemetic. In Sweden, patients would receive corticosteroids such as betamethasone (4–8 mg; 100% of patients) and an antiemetic such as tropisetron (5 mg; 100% of patients) before administration of doxorubicin/ifosfamide and dexamethasone (8 mg; 100% of patients) before administration of trabectedin. Patients would receive antiemetics and laxatives for 2-3 days after chemotherapy.

In all three countries a granulocyte-colony-stimulating factor (G-CSF, filgrastim 6 mg) would be administered to prevent neutropenia in ~65% of patients receiving doxorubicin/ifosfamide and ~15% of patients receiving gemcitabine-containing regimens. Other patients would not receive prophylactic G-CSF but would receive it therapeutically when they experience haematological toxicities.

All patients receiving an ifosfamide-containing chemotherapy would also receive mesna. Typically, the dose of mesna administered would be the same as the ifosfamide dose.

*Haematological Toxicities*. According to the interviewed oncologists, the main complications associated with the aforementioned regimens are grades 3-4 haematological toxicities (i.e., anaemia, thrombocytopenia, neutropenia, and febrile neutropenia). Hence, the healthcare costs associated with managing these complications have been incorporated into the model.

*Palliative Care*. According to the interviewees, palliative care can be introduced at any stage along the treatment pathway. The necessity for palliative care is guided by a patient's performance status and could be introduced even before the initiation of chemotherapy. Frequently, palliative care units work in collaboration with oncology services and provide patient care when the disease is too advanced, when patients are unable to receive chemotherapy, when patients experience difficult to control symptoms, or when there is no active treatment that is effective. Accordingly, the costs associated with palliative care have been incorporated into the model.

#### 2.2.2. Model Inputs: Clinical Outcomes

**Figure 2:**
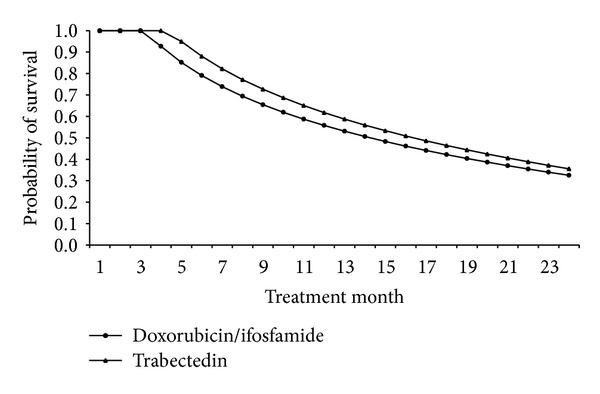
Survival rates associated with first-line treatment with doxorubicin/ifosfamide and trabectedin.

**Figure 3:**
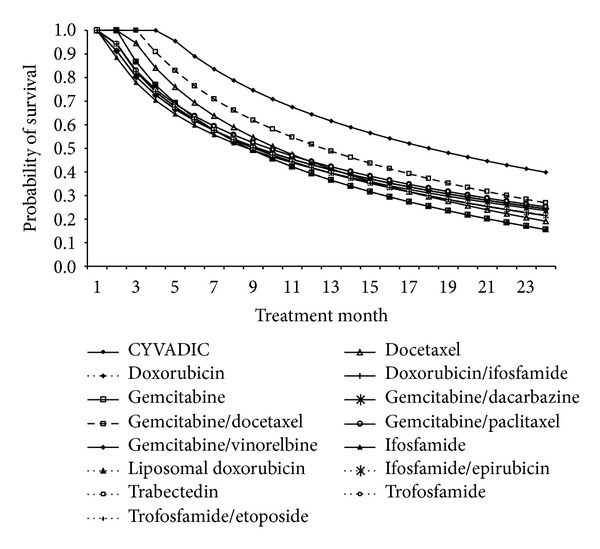
Survival rates associated with second-line treatments.

Clinical outcomes associated with the management of ASTS were estimated from the literature review. Published clinical outcomes analysed included the probability of achieving CR, PR, SD, and PD (Table 3), median duration of response (Table 3), cancer-related mortality stratified according to the regimens (Figures 2 and 3), and incidence of grades 3-4 haematological toxicities (Table 4). Where more than one publication was available, the mean rates were weighted according to the sample sizes.

The outcomes from studies in which doxorubicin/ifosfamide and trabectedin were used as first-line chemotherapies are shown separately from those studies in which these agents were used as second-line treatments. The literature review could not identify any publications reporting efficacy rates for second-line chemotherapy with CYVADIC (cyclophosphamide, vincristine, adriamycin, and dacarbazine), trofosfamide/etoposide, and gemcitabine/paclitaxel. Therefore, the efficacy rates for these cytotoxic agents were assumed to be the average of all the efficacy rates that were available for second-line chemotherapy (i.e., doxorubicin/ifosfamide, gemcitabine/docetaxel, and gemcitabine/dacarbazine). Also, efficacy rates for second-line chemotherapy with ifosfamide/epirubicin were assumed to be the same as those for doxorubicin/ifosfamide, as they were both ifosfamide-containing regimens, and the rates for liposomal doxorubicin were assumed to be the same as those for doxorubicin monotherapy since both are anthracyclines.

*Median Duration of Response*. Some publications reported only the overall median duration of response. Hence, the relationship between overall median duration of response and median duration associated with CR, PR, and SD derived from publications reporting stratified outcomes was used to estimate median duration of response for the missing response types. 

The literature review could not identify any publications reporting median duration of response following second-line chemotherapy with doxorubicin/ifosfamide, doxorubicin monotherapy, ifosfamide/epirubicin, gemcitabine/docetaxel, gemcitabine/paclitaxel, trofosfamide, trofosfamide/etoposide, CYVADIC, and liposomal doxorubicin monotherapy. Hence, the reported average median duration of response associated with second-line ifosfamide monotherapy, gemcitabine/dacarbazine, and gemcitabine monotherapy was used to estimate the median duration of response associated with these regimens, since the median duration of response was only available for these second-line regimens. It was decided to exclude trabectedin's duration of response from this extrapolation since it was the only new generation chemotherapeutic agent.

Table 3 summarises the probabilities of achieving one of the health states and the duration of remaining in a health state following first- and second-line chemotherapies that have been incorporated in the model.

*Mortality Rates*. Age-related mortality was estimated using published mortality rates [84]. The literature review was used to estimate cancer-related mortality rates. Using a least squares regression methodology, lines of best fit were derived to estimate cancer-related mortality rates at various time points. The resulting mortality curves were adjusted to exclude age-related mortality for Italy [84], Spain [84], and Sweden [84]. Cancer-related mortality rates were available for all first-line treatments [17, 18, 23, 24, 28, 29] and some second-line treatments: gemcitabine monotherapy [40, 43, 46, 47], ifosfamide monotherapy [38, 57, 60], trabectedin monotherapy [19, 34, 61, 63–65, 67], gemcitabine/vinorelbine [56], and docetaxel monotherapy [35, 37]. Cancer-related mortality rates could not be identified for the following second-line regimens: doxorubicin monotherapy, doxorubicin/ifosfamide, ifosfamide/epirubicin, gemcitabine/dacarbazine, gemcitabine/paclitaxel, CYVADIC, trofosfamide/etoposide, trofosfamide, and liposomal doxorubicin monotherapy. Hence, cancer-related mortality rates for doxorubicin monotherapy and liposomal doxorubicin monotherapy were assumed to be the same as those for second-line ifosfamide monotherapy, because they appear comparable in clinical practice (Table 3). Cancer-related mortality rates for doxorubicin/ifosfamide and ifosfamide/epirubicin were assumed to be the average of those for second-line ifosfamide monotherapy and trabectedin monotherapy because of the reported similarities in the average median duration of response between the regimens. Also the interviewed clinicians considered that the mortality rates associated with these three regimens were comparable in clinical practice. Cancer-related mortality rates for gemcitabine/paclitaxel, CYVADIC, trofosfamide, and trofosfamide/etoposide were assumed to be the average of those for second-line gemcitabine, ifosfamide, and trabectedin. This was based on the observed similarities in the average median duration of responses associated with the aforementioned regimens. 

The estimated survival rates following first-line treatment with doxorubicin/ifosfamide and trabectedin monotherapy that have been incorporated in the model are shown in Figure 2. The estimated survival rates following second-line treatment after failing first-line treatment with doxorubicin/ifosfamide and trabectedin monotherapy that have been incorporated in the model are shown in Figure 3.

*Incidence of Haematological Complications*. According to the interviewees only grades 3-4 haematological complications would result in additional healthcare resource utilisation. The incidence of haematological complications was estimated from the literature review. However, the review could not identify any publications reporting the incidence of grades 3-4 haematological complications following second-line treatment with doxorubicin/ifosfamide, gemcitabine/paclitaxel, CYVADIC, trofosfamide/etoposide, and ifosfamide/epirubicin. Consequently, the average of the available incidence rates associated with the second-line combination regimens was used.

Also not reported was the incidence of grades 3-4 haematological complications following second-line treatment with liposomal doxorubicin monotherapy. The relationship between the incidence rates associated with first-line doxorubicin monotherapy and liposomal doxorubicin monotherapy was used to estimate the missing incidence rates. This assumption was made on the basis that liposomal doxorubicin monotherapy has equivalent activity to doxorubicin monotherapy treatment [97]. Also, not reported was the incidence of anaemia following second-line treatment with doxorubicin monotherapy. Therefore, the average of the rates of anaemia associated with other second-line treatments was used to interpolate missing values.

Table 4 summarises the incidence of grades 3-4 haematological toxicities following first- and second-line treatments that have been incorporated in the model.

#### 2.2.3. Model Inputs: Utilities

Health state utilities for ASTS elicited from the general public using time trade-off methodology were assigned to the health states in our model [98]. The estimated utility values were as follows: complete response 0.60, partial response 0.51, stable disease 0.43, and progressive disease 0.30. 

#### 2.2.4. Model Outputs

By assigning unit costs in Euros at 2010/2011 prices (Table 5) to the resource use estimates in the different health states within the Markov model, the healthcare costs over two years after a patient initially received either doxorubicin/ifosfamide or trabectedin monotherapy were estimated. Unit costs that were only available for earlier periods were uprated to 2010/2011 prices using the relevant inflation index for each country.

The primary measure of clinical effectiveness in the model was the number of quality-adjusted life years (QALYs) two years after starting first-line treatment with doxorubicin/ifosfamide or trabectedin monotherapy. The model also estimated successful treatment at two years in terms of the proportion of patients achieving CR, PR, and SD.

In accordance with the guidelines for economic evaluations in Italy [99], Spain [100], and Sweden [101] healthcare costs and QALYs in the second year were each discounted at 3%.

### 2.3. Cost Effectiveness Analyses

The incremental cost effectiveness of doxorubicin/ifosfamide compared to trabectedin monotherapy was calculated as the difference between the expected discounted costs of the two treatment strategies over 2 years divided by the difference between the expected discounted number of QALYs of the two strategies over 2 years. Hence, the incremental cost effectiveness of doxorubicin/ifosfamide compared to trabectedin monotherapy was defined as the cost per QALY gained. If a treatment resulted in more QALYs for less cost, it was defined as a dominant treatment.

### 2.4. Sensitivity Analyses

Probabilistic sensitivity analyses (PSA) using Monte Carlo iterations (10,000 iterations of the model) were undertaken by simultaneously varying all the probabilities, utilities, unit costs, and resource use values within the model. The probabilities and utilities were varied randomly according to a beta distribution and the resource use estimates and unit costs were varied randomly according to a gamma distribution. Results from these analyses were used to construct cost effectiveness acceptability curves showing the probability of first-line treatment with doxorubicin/ifosfamide compared to trabectedin monotherapy to be cost effective at varying cost per QALY thresholds.

Deterministic sensitivity analyses were also performed to assess the impact of independently varying individual parameter values within the model. The parameter estimates were varied over plausible ranges by altering them to 20% below and 20% above the base case values.

## 3. Results

### 3.1. Expected Clinical Outcomes

The outcomes at two years following initial treatment with doxorubicin/ifosfamide or trabectedin are summarised in Table 6. Differences between the countries reflect the different second-line treatments that are used in Italy, Spain, and Sweden.

### 3.2. Expected Healthcare Costs

The expected costs at two years following initial treatment with doxorubicin/ifosfamide or trabectedin are summarised in Table 7. Differences between the countries reflect the different second-line treatments, different management algorithms, and different unit costs. Nevertheless, in all three countries, the expected two-year costs of starting treatment with doxorubicin/ifosfamide are between 4% and 10% less than those of starting treatment with trabectedin.

In Spain and Sweden the primary cost driver in patients starting chemotherapy with doxorubicin/ifosfamide was the cost of pre- and postchemotherapy medications. However, in Italy, the primary cost driver was the cost of second-line chemotherapy regimens. In all three countries, the primary cost driver in patients starting chemotherapy with trabectedin was the acquisition cost of this cytotoxic agent (Table 7).

### 3.3. Cost Effectiveness Analyses

Starting treatment with doxorubicin/ifosfamide instead of trabectedin monotherapy is expected to lead to a cost reduction of €1,710 in Italy, €3,497 in Spain, and €3,274 in Sweden. Additionally, starting treatment with doxorubicin/ifosfamide instead of trabectedin monotherapy is expected to lead to an improvement in health status at two years of 0.07 QALYs in Italy, 0.04 QALYs in Spain, and 0.02 QALYs in Sweden. Hence, doxorubicin/ifosfamide was found to be a dominant treatment relative to trabectedin in all three countries with a cost per QALY of −€26,308, −€87,423, and −€136,396 in Italy, Spain, and Sweden, respectively. 

### 3.4. Probabilistic Sensitivity Analyses

**Figure 4:**
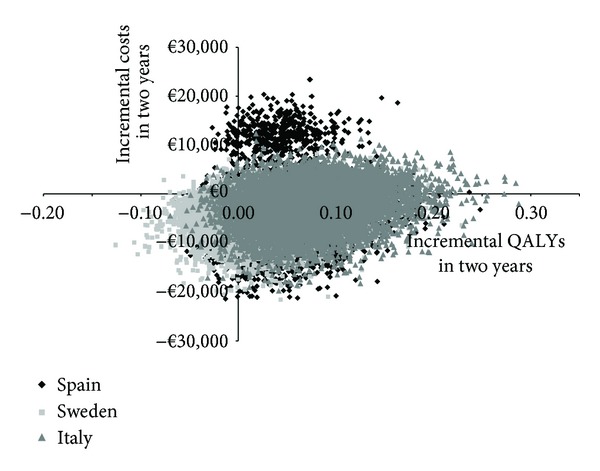
Scatterplot of the incremental cost effectiveness of doxorubicin/ifosfamide compared to trabectedin monotherapy (10,000 iterations of each model).

Probabilistic sensitivity analyses highlighted the distribution in the incremental costs and QALYs at two years (Figure 4), from which it can be seen that the majority of the samples are located in the dominant (bottom right) quadrant (Figure 4). These analyses also showed that there is greater dispersion in Spain and Sweden than in Italy.

**Figure 5:**
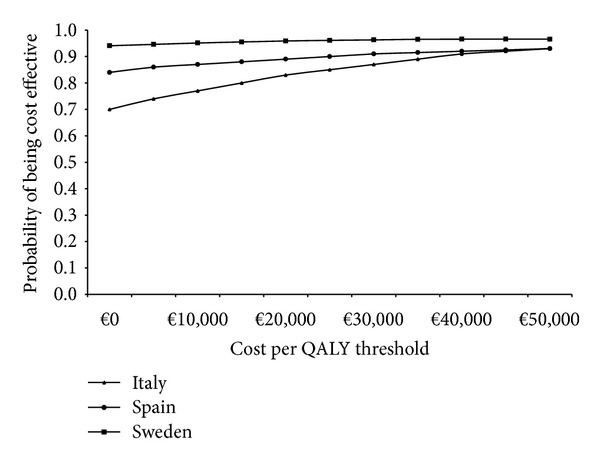
Acceptability curves.

Cost effectiveness acceptability curves generated from the probabilistic sensitivity analyses showed the probability of doxorubicin/ifosfamide to be cost effective compared to trabectedin monotherapy across a wide range of cost per QALY thresholds (Figure 5). At a threshold of €35,000 per QALY, >90% of a cohort would be cost effectively treated with doxorubicin/ifosfamide compared to trabectedin monotherapy in all three countries.

### 3.5. Deterministic Sensitivity Analyses

Extensive deterministic sensitivity analyses (Table 8) showed that the model is robust to plausible changes in the model inputs. Varying the model inputs between 20% below and 20% above the base case values showed that doxorubicin/ifosfamide remained a dominant treatment in Spain and Sweden and a cost-effective treatment in Italy, across all the variables.

Additionally, doxorubicin/ifosfamide remained a dominant treatment when the use of second-line treatments was excluded from the patients' pathways, by assuming that those who do not respond to first-line chemotherapy, or those with disease progression, only receive palliative care. In these circumstances, starting chemotherapy with doxorubicin/ifosfamide or trabectedin is expected to lead to a two-year cost of

- €14,567 and €32,858 per patient, respectively, in Italy,
- €18,085 and €26,198 per patient, respectively, in Spain,
- €21,385 and €23,410 per patient, respectively, in Sweden.

Additionally, starting chemotherapy with doxorubicin/ifosfamide or trabectedin is expected to lead to 0.274 QALYs and 0.178 QALYs per patient, respectively, at two years, irrespective of country.

## 4. Discussion

There have been several studies assessing the efficacy of first-line treatment of ASTS with trabectedin [18, 34]. Hence, the precedent had been set prior to performing this study to evaluate the cost effectiveness of doxorubicin/ifosfamide versus trabectedin as first-line treatment strategies for the management of this disease. Our literature search failed to find any health economic studies on ASTS in Italy, Spain, or Sweden. Consequently, using a range of published studies and estimates of resource use obtained from clinicians who manage sarcoma, a two-year Markov model was constructed to simulate the management of patients suffering from ASTS in each of these three countries. Due to the lack of published data, the time horizon of the model was limited to two years, by which time most patients would die. Markov models are suited to simulate the consequences of decisions when the timing of events is important and when events may happen more than once. Hence, they are appropriate for evaluating the consequences of decisions that are of a sequential or repetitive nature [102]. Since events such as response and relapse in ASTS recur over time, use of a Markov model was considered the most appropriate vehicle for performing this cost effectiveness analysis. 

There are potential limitations with the model, mainly due to the combination of numerous sources and data assumptions. The clinical basis of the model was diverse studies that included patients with different types of ASTS, different severity of disease, different age of sufferers, different administration schedules, and prior treatments. Therefore, the patient populations may not be identical in all the studies. Consequently, the clinical outcomes observed in this study may not necessarily reflect those observed in clinical practice. Also, the Markov model was based on many assumptions pertaining to cancer-related mortality, chemotherapy efficacy rates, and duration of response. These assumptions were necessary due to the limited availability of data pertaining to some of the regimens employed by the interviewed oncologists. Nevertheless, these assumptions were tested using extensive deterministic and probabilistic sensitivity analyses and found to be robust to changes in the model inputs. Notwithstanding this, there is potential for confounding in this study due to the lack of any direct comparative evidence between the two first-line treatment regimens and some of the second-line treatment efficacy estimates are based on assumptions. 

The literature search was unable to identify any published studies assessing healthcare resource utilisation and chemotherapy patterns for ASTS. Because of the low incidence of the disease, healthcare resource utilisation was not collected prospectively but was estimated from interviews with six oncologists in each country. Consequently, resource use for the “average clinician” throughout each country may not be the same as that for those clinicians who participated in this study. 

The interviewees indicated that there are no treatment guidelines for the management of ASTS, and in Sweden, only ~20–30% of patients are covered by the Scandinavian Sarcoma Group protocol (SSG XX) [103]. Therefore, the chemotherapy patterns in this study reflect the individual judgment of the oncologists interviewed. Consequently, the levels of healthcare resource utilisation observed in the analysis might not be indicative for each country as a whole. Also, as a consequence, it is not known how the study results would generalise to patients treated in other oncology centres. Moreover, treatment of ASTS is very individual and the type of regimen chosen depends on (1) the histological sub-type of sarcoma and (2) the patient's characteristics. Also, following treatment failure, a proportion of patients would be enrolled into clinical trials or would only receive palliative care. Furthermore, treatment patterns are not very standardised. Hence, there may be other treatments that are used but have not been mentioned by the interviewees. Consequently, it was a very challenging task for the interviewees to provide generalised treatment patterns. Nevertheless, the chemotherapy patterns presented in this study provide an overview of current practice in all three countries. Moreover, according to the probabilistic sensitivity analyses, the conclusions reached are robust to changes in the distribution of the second-line treatments.

The model incorporated resource use and utility values for an “average patient” and did not take into account stage of disease and patients' characteristics such as age, gender, suitability of patients for different chemotherapy regimens, and other comorbidities. The model considered only direct healthcare costs borne by the secondary healthcare sector in each country and did not consider costs borne by the community. Moreover, the costs and consequences of managing patients who survive beyond two years are also excluded. Also, the study excluded costs incurred by patients, families, and/or their caregivers and indirect costs incurred by society as a result of patients taking time off work and/or not being able to lead productive lives, although the majority of patients are expected to have a mean age of 65 years. Consequently, inclusion of these costs may affect the study's results and need to be studied further in larger populations.

First-line treatment with doxorubicin/ifosfamide was found to be cost effective when compared to first-line trabectedin monotherapy in Italy, Spain, and Sweden. In this study, patients' health status, in terms of the number of QALYs at two years, is a reflection of the probability of being in different health states over the study period and the duration of being in each health state. According to the Markov model, first-line treatment with doxorubicin/ifosfamide yields more QALYs than with trabectedin monotherapy, irrespective of whether second-line chemotherapy is included in the analysis. Additionally, in all three countries use of doxorubicin/ifosfamide leads to lower two-year healthcare costs. Moreover, at a threshold of €35,000 per QALY, >90% of a cohort is expected to be cost effectively treated with doxorubicin/ifosfamide compared to trabectedin monotherapy in all three countries. The primary cost driver of managing patients in the trabectedin monotherapy group is the unit cost of this cytotoxic agent. Subsequent to completion of this study the results of the landmark EORTC62012 study comparing doxorubicin with doxorubicin/ifosfamide as first-line treatment for ASTS have been reported as an abstract [104]. This multi-institutional, phase III study recruited 455 patients and demonstrated an improved progression-free survival for the combination arm but significantly worse toxicity and no overall survival benefit. Many oncologists would therefore consider single agent doxorubicin to be the new standard of care, a treatment that would be expected to have lower acquisition and toxicity management costs than doxorubicin/ifosfamide. Hence, it is difficult to see how the high acquisition cost of trabectedin affords value for money to the publicly funded healthcare systems in Italy, Spain, and Sweden when used as a first-line treatment for ASTS. Consequently, trabectedin should be used following failure of doxorubicin and ifosfamide treatment in accordance with its indication.

In the absence of any published health economic studies assessing the cost effectiveness of treatments for the management of ASTS in any country, it is not known how the results of the present analysis would generalise to other settings and patient groups and whether all important factors for the decision under consideration have been taken into account. Nevertheless, within the limitations of the present study, doxorubicin/ifosfamide (or single agent doxorubicin [104]) is expected to be a preferred first-line treatment strategy for the management of ASTS compared to trabectedin monotherapy in all three countries.

In conclusion, within the model's limitations, first-line treatment of patients with ASTS with doxorubicin/ifosfamide instead of trabectedin monotherapy affords a cost-effective use of publicly funded healthcare resources in Italy, Spain, and Sweden. These findings support the recommendation that trabectedin should remain a second/third-line treatment.

## Figures and Tables

**Table 1:** Probabilities of receiving second-line treatments.

Regimen	Probability of receiving second-line treatment in		
	Italy	Spain	Sweden
Following first-line treatment with doxorubicin/ifosfamide			
CYVADIC^*∧*^	<0.01	<0.01	0.10
Gemcitabine/dacarbazine	<0.01	0.12	<0.01
Gemcitabine/docetaxel	0.18	0.20	0.48
Gemcitabine/paclitaxel	<0.01	0.10	<0.01
Gemcitabine/vinorelbine	<0.01	0.08	<0.01
Gemcitabine monotherapy	<0.01	0.12	<0.01
Ifosfamide monotherapy	0.20	0.12	<0.01
Liposomal doxorubicin	0.12	<0.01	<0.01
Trofosfamide	<0.01	<0.01	0.12
Trabectedin monotherapy	0.50	0.26	0.30
Following first-line treatment with trabectedin monotherapy			
Docetaxel monotherapy	0.26	<0.01	<0.01
Doxorubicin/ifosfamide	<0.01	<0.01	0.67
Doxorubicin monotherapy	<0.01	<0.01	<0.01
Gemcitabine/docetaxel	0.05	0.44	0.25
Ifosfamide/epirubicin	0.16	<0.01	<0.01
Ifosfamide monotherapy	0.53	0.56	<0.01
Trofosfamide/etoposide	<0.01	<0.01	0.08

^*∧*^CYVADIC: cyclophosphamide, vincristine, adriamycin, and dacarbazine.

**Table 2:** Characteristics of chemotherapy regimens incorporated into the model.

Regimen	Mean dose per cycle	Admissions/outpatient clinic attendances per cycle
CYVADIC^*∧*^	600 mg/m^2^ cyclophosphamide	4 outpatient clinic attendances
	1 mg/m^2^ vincristine	
	30 mg/m^2^ doxorubicin	
	250 mg/m^2^ dacarbazine	
Docetaxel	100 mg/m^2 ^docetaxel	1 outpatient clinic attendance
Doxorubicin	75 mg/m^2^ doxorubicin	1 outpatient clinic attendance
Doxorubicin/ifosfamide	66 mg/m^2^ doxorubicin	3-4-day admission
	8.5 g/m^2^ ifosfamide	
Ifosfamide	12.5 g/m^2^ ifosfamide	4-day admission or 2 outpatient clinic attendances
Ifosfamide/epirubicin	100 mg/m^2^ epirubicin	3-day admission
	5 g/m^2^ ifosfamide	
Gemcitabine	1,000 mg/m^2^ gemcitabine	2 outpatient clinic attendances
Gemcitabine/dacarbazine	1,766 mg/m^2^ gemcitabine	2 outpatient clinic attendances
	700 mg/m^2 ^dacarbazine	
Gemcitabine/docetaxel	1,000 mg/m^2^ gemcitabine	2 outpatient clinic attendances
	75 mg/m^2^ docetaxel	
Gemcitabine/paclitaxel	1,000 mg/m^2^ gemcitabine	2 outpatient clinic attendances
	125 mg/m^2^ paclitaxel	
Gemcitabine/vinorelbine	1,250 mg/m^2^ gemcitabine	2 outpatient clinic attendances
	25 mg/m^2^ vinorelbine	
Liposomal doxorubicin	50 mg/m^2 ^doxorubicin	1 outpatient clinic attendance
Trabectedin	1.3 mg/m^2 ^trabectedin	2-day admission
Trofosfamide	200 mg/m^2 ^trofosfamide	Oral administration over ~10 days, no hospital attendance
Trofosfamide/etoposide	150 mg/m^2^ trofosfamide	Oral administration over ~10 days, no hospital attendance
	25 mg/m^2^ etoposide	

^*∧*^CYVADIC: cyclophosphamide, vincristine, adriamycin, and dacarbazine.

**Table 3:** Efficacy rates and duration of response associated with different chemotherapy regimens for ASTS.

	Probability of achieving:				Median duration of response (months) in:		
	Complete remission	Partial remission	Stable disease	Progressive disease	Complete remission	Partial remission	Stable disease
First-line treatments							
Doxorubicin/ifosfamide	0.06	0.21	0.38	0.35	15.44	7.69	6.41
	[16, 17, 22–32]	[16, 17, 22–32]	[16, 17, 22–32]	[16, 17, 22–32]	[16, 17, 22, 24, 27, 28, 31, 33]	[16, 17, 22, 24, 27, 28, 31, 33]	[16, 17, 22, 24, 27, 28, 31, 33]
Trabectedin	0.03	0.11	0.14	0.72	17.74	8.75	7.48
	[34]	[34]	[34]	[34]	[34]	[34]	[34]
Second-line treatments							
CYVADIC^∗*∧*^	0.03	0.19	0.39	0.39	12.13	6.57	5.75
Docetaxel	0.00	0.11	0.25	0.64	0.00	6.60	7.17
	[35–37]	[35–37]	[35–37]	[35–37]	[35, 36]	[35, 36]	[35, 36]
Doxorubicin	0.02	0.07	0.31	0.61	12.13*	6.57*	5.75*
	[38, 39]	[38, 39]	[38, 39]	[38, 39]			
Doxorubicin/ifosfamide	0.05	0.27	0.37	0.31	12.13*	6.57*	5.75*
	[22, 38]	[22, 38]	[22, 38]	[22, 38]			
Gemcitabine	0.00	0.08	0.33	0.59	0.00	4.46	3.86
	[40–45]	[40–45]	[40–45]	[40–45]	[43, 46, 47]	[43, 46, 47]	[43, 46, 47]
Gemcitabine/dacarbazine	0.01	0.10	0.39	0.51	10.48	6.50	5.79
	[48–50]	[48–50]	[48–50]	[48–50]	[48]	[48]	[48]
Gemcitabine/docetaxel	0.05	0.19	0.41	0.35	12.13*	6.57*	5.75*
	[40, 51–54]	[40, 51–54]	[40, 51–54]	[40, 51–54]			
Gemcitabine/paclitaxel*	0.03	0.19	0.39	0.39	12.13	6.57	5.75
Gemcitabine/vinorelbine	0.02	0.10	0.10	0.78	16.10	16.10	9.60
	[55, 56]	[55, 56]	[55, 56]	[55, 56]	[56]	[56]	[56]
Ifosfamide	0.02	0.13	0.24	0.61	13.77	8.75	7.61
	[38, 57–60]	[38, 57–60]	[38, 57–60]	[38, 57–60]	[58, 60]	[58, 60]	[58, 60]
Ifosfamide/epirubicin*	0.05	0.27	0.37	0.31	12.13	6.57	5.75
Liposomal doxorubicin*	0.02	0.07	0.31	0.61	12.13	6.57	5.75
Trabectedin	<0.01	0.07	0.44	0.49	16.14	10.25	8.91
	[19, 34, 61–67]	[19, 34, 61–67]	[19, 34, 61–67]	[19, 34, 61–67]	[19, 34, 61, 63–65]	[19, 34, 61, 63–65]	[19, 34, 61, 63–65]
Trofosfamide	0.00	0.03	0.19	0.79	12.13*	6.57*	5.75*
	[68–70]	[68–70]	[68–70]	[68–70]			
Trofosfamide/etoposide*	0.03	0.19	0.39	0.39	12.13	6.57	5.75

*Values were estimated. ^*∧*^CYVADIC: cyclophosphamide, vincristine, adriamycin and dacarbazine.

**Table 4:** Probabilities of patients developing haematological toxicities stratified by chemotherapy regimen.

	Probability of developing			
	neutropenia	febrile neutropenia	thrombocytopenia	anaemia
First-line treatments				
Doxorubicin/ifosfamide	0.82	0.12	0.23	0.35
	[23, 24, 26, 30]	[24, 26]	[17, 23, 24, 26, 30]	[23, 26, 30]
Trabectedin	0.33	0.00	0.00	0.03
	[34]	[34]	[34]	[34]
Second-line treatments				
CYVADIC^∗*∧*^	0.52	0.19	0.17	0.16
Docetaxel	0.90	0.12	0.03	0.08
	[36, 37]	[35, 37]	[35–37]	[35–37]
Doxorubicin	0.84	0.19	0.09	0.18*
	[39]	[39]	[39]	
Doxorubicin/ifosfamide*	0.52	0.19	0.17	0.18
Gemcitabine	0.18	0.07	0.18	0.11
	[40, 41, 43, 47, 71]	[33, 40, 41, 43, 71]	[33, 40–43, 46, 71]	[40–43, 71]
Gemcitabine/dacarbazine	0.46 [49]	0.19*	0.12 [49]	0.23 [49]
Gemcitabine/docetaxel	0.31	0.09	0.33	0.18
	[52, 54]	[52, 54]	[40, 52, 54]	[52, 54]
Gemcitabine/paclitaxel*	0.52	0.19	0.17	0.16
Gemcitabine/vinorelbine	0.38 [56]	0.08 [56]	0.10 [56]	0.05 [56]
Ifosfamide	0.82	0.39	0.13	0.12
	[57–60]	[58, 59]	[57–60]	[57–59]
Ifosfamide/epirubicin*	0.52	0.19	0.17	0.18
Liposomal doxorubicin*	0.07	0.02	0.00	0.35
Trabectedin	0.50	0.06	0.16	0.18
	[19, 34, 61, 64, 65, 67]	[19, 34, 61, 65]	[19, 34, 61, 64, 65, 67]	[19, 34, 61, 65]
Trofosfamide	0.52*	0.19*	0.17*	0.25 [70]
Trofosfamide/etoposide*	0.52	0.19	0.17	0.16

^*∧*^CYVADIC: cyclophosphamide, vincristine, adriamycin, and dacarbazine.

*Values were estimated.

**Table 5:** Unit resource costs (in Euros at 2010/2011 prices) used in the model.

Resource	Unit costs (in Euros at 2010/2011 prices)					
	Italy		Spain		Sweden	
Aprepitant (125 mg)			€90.9	[72]	€63.8	[73]
Betapred (4 mg)					€6.4	[73]
Betamethasone (8 mg)					€3.2	[73]
Biopsy	€129.1	[74]	€603.7	[75]	€314.1	[76]
Bone scintigraphy			€296.8	[77]		
Chest X-ray	€16.2	[78]	€6.5	[79]	€48.7	[76]
CT scan	€86.3	[74]	€87.5	[79]	€313.6	[76]
Cyclophosphamide (200 mg)					€4.1	[80]
Dacarbazine (1000 mg)			€21.7	[80]		
Dacarbazine (200 mg)					€8.9	[80]
Dexamethasone (0.75 mg, 10 tablets)	€1.1	[81]				
Dexamethasone (1 mg, 30 tablets)			€3.0	[72]		
Diphenhydramine (25 mg, 25 capsules)			€1.4	[72]		
Docetaxel (10 mg)	€84.4	[80]				
Docetaxel (100 mg)			€182.8	[80]		
Docetaxel (80 mg)					€403.1	[80]
Doxorubicin (50 mg)	€119.5	[80]	€4.1	[80]	€59.8	[80]
Echocardiography	€51.7	[74]	€18.2	[79]	€214.9	[76]
Electrocardiogram	€13.0	[78]	€13.5	[82]	€334.2	[76]
Epirubicin (50 mg)	€81.2	[80]				
Etoposide (100 mg)					€20.8	[80]
Filgrastim (300 mcg)			€94.8	[72]		
Filgrastim (6 mg)	€149.8	[81]				
Gemcitabine (1000 mg)	€113.2	[80]	€75.7	[80]	€104.6	[80]
General surgeon consultation					€230.3	[76]
Granisetron (1 mg, 10 tablets)	€133.9	[81]	€48.1	[72]		
Haematology tests	€3.7	[83]	€20.5	[82]	€5.2	[76]
Hospitalisation for chemotherapy infusion/day	€238.3	[84]	€212.9	[84]	€288.6	[84]
Ifosfamide (1 g)	€30.7	[80]	€19.7	[80]		
Ifosfamide (2 g)					€65.7	[80]
Lenograstim (1 vial)	€153.4	[81]				
Levocetirizine (5 mg, 20 tablets)	€10.5	[81]				
Liver function test	€9.2	[83]	€11.7	[79]		
Liposomal doxorubicin (2 mg)	€548.2	[80]				
Managing anaemia	€1,354.8	[85]	€900.0	[86]	€548.6	[87]
Managing febrile neutropenia	€3,305.0	[88]	€3829.5	[89]	€2,892.0	[90]
Managing neutropenia	€523.3	[85]	€2086.1	[91]		
Managing thrombocytopenia	€1,354.8	[85]	€900.0	[86]	€548.6	[87]
Mesna (3 g)			€13.2	[72]		
Mesna (6 g)	€25.7	[81]				
Mesna (5 g)					€192.2	[73]
Metoclopramide (250 mL)			€2.7	[72]		
MRI scan	€285.8	[83]	€168.0	[79]	€386.4	[76]
Multidisciplinary team assessment	€48.7	[74]	€61.3	[79]	€1,816.5	[76]
Nuclear medicine specialist consultation			€61.5	[79]		
Nurse home visit	€51.2	[92]	€56.5	[77]		
Oncologist consultation	€21.6	[83]	€61.5	[79]	€283.7	[76]
Ondansetron (4 mg, 6 tablets)	€57.8	[81]				
Ondansetron (4 mg, 15 tablets)			€36.3	[72]		
Orthopaedic surgeon consultation	€21.6	[83]	€61.5	[79]	€102.4	[76]
Outpatient attendance for chemotherapy	€122.8	[84]	€98.9	[84]	€288.6	[84]
Paclitaxel (30 mg)			€83.8	[80]		
Palliative care per patient	€3,265.0	[93]	€2167.7	[94]	€1,343.9	[95, 96]
Palonosetron (250 mcg)			€104.6	[72]		
Pathologist consultation	€21.6	[83]	€61.5	[79]		
Pegfilgrastim (1 syringe)			€1,062.6	[72]	€1,322.5	[73]
PET scan	€1,071.7	[74]	€500.0	[79]	€314.1	[76]
Radiologist consultation	€21.6	[83]	€61.5	[79]		
Radiotherapist consultation	€21.6	[83]	€61.5	[79]		
Renal function test	€5.0	[83]	€8.9	[79]		
Secondary care hospital specialist visit			€61.5	[79]		
Trabectedin (1 mg, 1 vial)	€2,970.1	[80]	€2,049.9	[80]	€1,913.3	[80]
Trofosfamide 50 mg/m^2^					€1.4	[80]
Tropisetron (5 mg)					€20.5	[73]
Ultrasound scan	€17.6	[74]	€18.2	[79]		
Urine analysis	€6.1	[83]	€1.8	[79]	€20.9	[76]
Vincristine (1 mg)					€16.2	[80]
Vinorelbine (1 mL)			€24.1	[80]		

In Sweden unit costs were converted from Swedish Krona (SEK) to Euros at the rate of €1 = 9.55 SEK.

**Table 6:** Clinical outcomes at two years.

	Italy		Spain		Sweden	
	Doxorubicin/ifosfamide	Trabectedin	Doxorubicin/ifosfamide	Trabectedin	Doxorubicin/ifosfamide	Trabectedin
Probability of						
complete response	0.01	<0.01	<0.01	<0.01	<0.01	0.01
partial response	0.01	<0.01	0.01	<0.01	0.01	0.01
stable disease	0.02	0.01	0.02	0.01	0.02	0.01
progressive disease	0.54	0.51	0.54	0.53	0.54	0.59
dying	0.43	0.47	0.42	0.45	0.42	0.39
Number of QALYs per patient	0.595 (0.593, 0.597)	0.530 (0.528, 0.533)	0.590 (0.587, 0.593)	0.550 (0.547, 0.553)	0.608 (0.606, 0.611)	0.584 (0.582, 0.587)

95% confidence intervals in parentheses.

**Table 7:** Expected healthcare costs (at 2010/2011 prices) over 2 years following first-line treatment with doxorubicin/ifosfamide combination and trabectedin monotherapy.

Resource	Expected healthcare costs per patient (Euros at 2010/2011 prices) over 2 years following first-line treatment											
	Italy				Spain				Sweden			
	Doxorubicin/ifosfamide		Trabectedin		Doxorubicin/ifosfamide		Trabectedin		Doxorubicin/ifosfamide		Trabectedin	
Diagnosis	€634.4	(2%)	€634.4	(2%)	€1886.5	(6%)	€1886.5	(6%)	€2416.8	(7%)	€2416.8	(6%)
First-line cytotoxics	€2302.9	(6%)	€26885.4	(66%)	€1491.8	(5%)	€18432.1	(54%)	€3172.2	(9%)	€17934.5	(45%)
Second-line cytotoxics	€17007.3	(44%)	€2556.9	(6%)	€6524.1	(21%)	€1761.6	(5%)	€8469.5	(23%)	€3224.8	(8%)
Evaluations of response	€2025.7	(5%)	€1641.7	(4%)	€1280.4	(4%)	€1098.7	(3%)	€2758.0	(8%)	€2453.2	(6%)
Hospitalisations for chemotherapy infusion	€5093.0	(13%)	€3765.6	(9%)	€4704.7	(15%)	€3564.3	(10%)	€4326.8	(12%)	€3685.2	(9%)
Outpatient attendances for chemotherapy	€291.3	(<1%)	€338.0	(1%)	€560.9	(2%)	€423.4	(1%)	€998.6	(3%)	€439.0	(1%)
Tests before each cycle of chemotherapy	€212.5	(<1%)	€118.0	(<1%)	€266.2	(1%)	€197.9	(1%)	€244.3	(1%)	€217.0	(1%)
Pre- and postchemotherapy medication	€5706.9	(15%)	€1107.3	(3%)	€7621.1	(25%)	€1665.8	(5%)	€11732.4	(32%)	€7458.6	(19%)
Palliative care	€2918.9	(8%)	€1773.3	(4%)	€1942.7	(6%)	€1932.3	(6%)	€1200.9	(3%)	€1265.9	(3%)
Management of haematological toxicity	€2728.6	(7%)	€1811.1	(4%)	€4421.0	(14%)	€3233.7	(9%)	€1187.2	(3%)	€685.2	(2%)

Total	€38921.7	(100%)	€40631.7	(100%)	€30699.4	(100%)	€34196.3	(100%)	€36506.7	(100%)	€39780.2	(100%)

(Percentage of total expected cost is in parenthesis).

**Table 8:** Sensitivity analyses.

Scenario	Base case value in Italy	Base case value in Spain	Base case value in Sweden	Effect
Duration of partial remission following first-line treatment with doxorubicin/ifosfamide ranges from 6.1 to 9.2 months	7.7 months	7.7 months	7.7 months	Doxorubicin/ifosfamide remains a dominant treatment
Duration of stable disease following first-line treatment with doxorubicin/ifosfamide ranges from 5.1 to 7.7 months	6.4 months	6.4 months	6.4 months	Doxorubicin/ifosfamide remains a dominant treatment
Duration of partial remission following first-line treatment with trabectedin ranges from 7.0 to 10.6 months	8.8 months	8.8 months	8.8 months	Doxorubicin/ifosfamide remains a dominant treatment
Duration of stable disease following first-line treatment with trabectedin ranges from 6.0 to 9.0 months	7.5 months	7.5 months	7.5 months	Doxorubicin/ifosfamide remains a dominant treatment
Probability of being in stable disease after first-line doxorubicin/ifosfamide ranges from 0.3 to 0.5	0.38	0.38	0.38	Doxorubicin/ifosfamide remains a dominant treatment
Probability of being in stable disease after first-line trabectedin ranges from 0.1 to 0.2	0.14	0.14	0.14	Doxorubicin/ifosfamide remains a dominant treatment
Probability of being in stable disease after second-line trabectedin ranges from 0.35 to 0.50	0.44	0.44	0.44	Doxorubicin/ifosfamide remains a dominant treatment
Probability of switching to trabectedin after first-line doxorubicin/ifosfamide ranges from 80% below to 20% above the base case value	0.50	0.26	0.30	Doxorubicin/ifosfamide remains a dominant treatment except in Italy where its costeffectiveness ranges from being dominant to €21,500 per QALY, breaking even at a probability of 0.55
Length of hospital stay for doxorubicin/ifosfamide infusion ranges from 1 to 5 days	3 days	4 days	3 days	Doxorubicin/ifosfamide remains a dominant treatment except in Italy where its costeffectiveness ranges from being dominant to €16,400 per QALY, breaking even at 4 days
Unit cost of doxorubicin ranges from 80% below to 20% above the base case value	€119.50	€4.11	€59.79	Doxorubicin/ifosfamide remains a dominant treatment
Unit cost of ifosfamide ranges from 80% below to 20% above the base case value	€30.71	€19.71	€65.65	Doxorubicin/ifosfamide remains a dominant treatment
Unit cost of trabectedin ranges from 80% below to 20% above the base case value	€2,970.10	€2,049.91	€1,913.29	Doxorubicin/ifosfamide remains a dominant treatment except in Italy where its costeffectiveness ranges from €11,200 per QALY to being dominant, breaking even at €2,570
Cost of managing adverse events ranges from 80% below to 20% above the base case values				Doxorubicin/ifosfamide remains a dominant treatment
Cost of pre- and postchemotherapy medications ranges from 80% below to 20% above the base case values				Doxorubicin/ifosfamide remains a dominant treatment
Cost of palliative care ranges from 80% below to 20% above the base case values				Doxorubicin/ifosfamide remains a dominant treatment
Utility for progressive disease ranges from 0.24 to 0.36	0.30	0.30	0.30	Doxorubicin/ifosfamide remains a dominant treatment
Utility for stable disease ranges from 0.34 to 0.52	0.43	0.43	0.43	Doxorubicin/ifosfamide remains a dominant treatment
Difference in QALYs gained following the start of treatment with doxorubicin/ifosfamide and trabectedin ranges from 80% below and 20% above the base case value	0.07	0.04	0.02	Doxorubicin/ifosfamide remains a dominant treatment
